# 1284. Occurrence of β-Lactamases among *Enterobacterales* Isolated from United States Hospitals: Results of the INFORM Surveillance Program for Ceftazidime-Avibactam

**DOI:** 10.1093/ofid/ofab466.1476

**Published:** 2021-12-04

**Authors:** Mariana Castanheira, Mariana Castanheira, Lalitagauri M Deshpande, Timothy B Doyle, Rodrigo E Mendes, Helio S Sader

**Affiliations:** JMI Laboratories, North Liberty, IA

## Abstract

**Background:**

Carbapenems are broadly used for the treatment of ESBL-producing *Enterobacterales* isolates. The use of these agents led to an increase of carbapenem resistance among *Enterobacterales*. Monitoring isolates that carry β-lactamases is important to understand their prevalence and susceptibility to clinically available antimicrobial agents. We evaluated the prevalence of β-lactamases and the activity of antimicrobial agents against 1,209 isolates collected in 69 US hospitals.

**Methods:**

A total of 9,686 *Enterobacterales* isolates collected during 2019 were susceptibility (S) tested by reference broth microdilution methods. Isolates submitted to whole genome sequencing were: (1) *Escherichia coli* (EC) and *Klebsiella pneumoniae* (KPN; n=817) displaying MIC values ≥2 mg/L for at least 2 of the following β-lactams: ceftazidime, ceftriaxone, aztreonam, or cefepime; (2) *Enterobacter cloacae* (ECL) and *Citrobacter* spp. (CIT; n=351) displaying MIC values ≥16 mg/L for ceftazidime and/or ≥2 mg/L for cefepime; and (3) *Enterobacterales* (n=118) displaying elevated carbapenem (meropenem and/or imipenem) MIC results at >1 mg/L.

**Results:**

A total of 723 isolates harbored ESBL genes but did not carry carbapenemases. The most common ESBL gene was *bla*_CTX-M-15_ (n=516), followed by *bla*_CTX-M-14_ (n=153). Most of these isolates were EC (278/147 for *bla*_CTX-M-15_/*bla*_CTX-M-14_), but 220 KPN harbored *bla*_CTX-M-15_. A total of 302 EC and KPN isolates carried *bla*_OXA-1_. Among ECL and CIT, *bla*_CTX-M-15_ and SHV genes encoding ESBLs were noted among 18 and 18 isolates. Carbapenemase genes were noted among 77 isolates, including 65 *bla*_KPC_, 3 *bla*_SME_, 6 *bla*_OXA-48_-like, and 3 *bla*_NDM_. Ceftazidime-avibactam (CAZ-AVI) was the only agent active against all ESBL-producers that did not carry carbapenemases (Table). CAZ-AVI was active against 90.9% of the isolates producing carbapenemases. Isolates resistant to this combination included 3 NDM-producers and 1 isolate harboring *bla*_KPC-31_.

**Conclusion:**

*Enterobacterales* isolates carrying ESBLs, mainly *bla*_CTX-M-15_, were very prevalent in this collection of US isolates. CAZ-AVI was very active against isolates tested, including isolates producing carbapenemases that displayed resistance to many comparator agents.

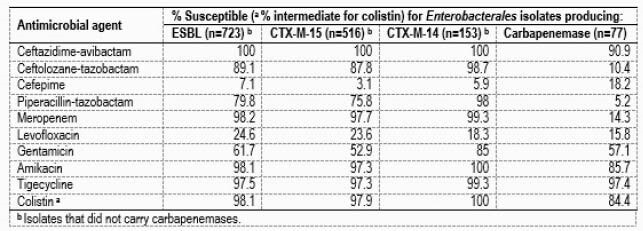

**Disclosures:**

**Mariana Castanheira, PhD**, **AbbVie (formerly Allergan**) (Research Grant or Support)**Bravos Biosciences** (Research Grant or Support)**Cidara Therapeutics, Inc.** (Research Grant or Support)**Cipla Therapeutics** (Research Grant or Support)**Cipla USA Inc.** (Research Grant or Support)**GlaxoSmithKline** (Research Grant or Support)**Melinta Therapeutics, Inc.** (Research Grant or Support)**Melinta Therapeutics, LLC** (Research Grant or Support)**Pfizer, Inc.** (Research Grant or Support)**Qpex Biopharma** (Research Grant or Support)**Shionogi** (Research Grant or Support)**Spero Therapeutics** (Research Grant or Support) **Mariana Castanheira, PhD**, Affinity Biosensors (Individual(s) Involved: Self): Research Grant or Support; Allergan (Individual(s) Involved: Self): Research Grant or Support; Amicrobe, Inc (Individual(s) Involved: Self): Research Grant or Support; Amplyx Pharma (Individual(s) Involved: Self): Research Grant or Support; Artugen Therapeutics USA, Inc. (Individual(s) Involved: Self): Research Grant or Support; Astellas (Individual(s) Involved: Self): Research Grant or Support; Basilea (Individual(s) Involved: Self): Research Grant or Support; Beth Israel Deaconess Medical Center (Individual(s) Involved: Self): Research Grant or Support; BIDMC (Individual(s) Involved: Self): Research Grant or Support; bioMerieux Inc. (Individual(s) Involved: Self): Research Grant or Support; BioVersys Ag (Individual(s) Involved: Self): Research Grant or Support; Bugworks (Individual(s) Involved: Self): Research Grant or Support; Cidara (Individual(s) Involved: Self): Research Grant or Support; Cipla (Individual(s) Involved: Self): Research Grant or Support; Contrafect (Individual(s) Involved: Self): Research Grant or Support; Cormedix (Individual(s) Involved: Self): Research Grant or Support; Crestone, Inc. (Individual(s) Involved: Self): Research Grant or Support; Curza (Individual(s) Involved: Self): Research Grant or Support; CXC7 (Individual(s) Involved: Self): Research Grant or Support; Entasis (Individual(s) Involved: Self): Research Grant or Support; Fedora Pharmaceutical (Individual(s) Involved: Self): Research Grant or Support; Fimbrion Therapeutics (Individual(s) Involved: Self): Research Grant or Support; Fox Chase (Individual(s) Involved: Self): Research Grant or Support; GlaxoSmithKline (Individual(s) Involved: Self): Research Grant or Support; Guardian Therapeutics (Individual(s) Involved: Self): Research Grant or Support; Hardy Diagnostics (Individual(s) Involved: Self): Research Grant or Support; IHMA (Individual(s) Involved: Self): Research Grant or Support; Janssen Research & Development (Individual(s) Involved: Self): Research Grant or Support; Johnson & Johnson (Individual(s) Involved: Self): Research Grant or Support; Kaleido Biosceinces (Individual(s) Involved: Self): Research Grant or Support; KBP Biosciences (Individual(s) Involved: Self): Research Grant or Support; Luminex (Individual(s) Involved: Self): Research Grant or Support; Matrivax (Individual(s) Involved: Self): Research Grant or Support; Mayo Clinic (Individual(s) Involved: Self): Research Grant or Support; Medpace (Individual(s) Involved: Self): Research Grant or Support; Meiji Seika Pharma Co., Ltd. (Individual(s) Involved: Self): Research Grant or Support; Melinta (Individual(s) Involved: Self): Research Grant or Support; Menarini (Individual(s) Involved: Self): Research Grant or Support; Merck (Individual(s) Involved: Self): Research Grant or Support; Meridian Bioscience Inc. (Individual(s) Involved: Self): Research Grant or Support; Micromyx (Individual(s) Involved: Self): Research Grant or Support; MicuRx (Individual(s) Involved: Self): Research Grant or Support; N8 Medical (Individual(s) Involved: Self): Research Grant or Support; Nabriva (Individual(s) Involved: Self): Research Grant or Support; National Institutes of Health (Individual(s) Involved: Self): Research Grant or Support; National University of Singapore (Individual(s) Involved: Self): Research Grant or Support; North Bristol NHS Trust (Individual(s) Involved: Self): Research Grant or Support; Novome Biotechnologies (Individual(s) Involved: Self): Research Grant or Support; Paratek (Individual(s) Involved: Self): Research Grant or Support; Pfizer (Individual(s) Involved: Self): Research Grant or Support; Prokaryotics Inc. (Individual(s) Involved: Self): Research Grant or Support; QPEX Biopharma (Individual(s) Involved: Self): Research Grant or Support; Rhode Island Hospital (Individual(s) Involved: Self): Research Grant or Support; RIHML (Individual(s) Involved: Self): Research Grant or Support; Roche (Individual(s) Involved: Self): Research Grant or Support; Roivant (Individual(s) Involved: Self): Research Grant or Support; Salvat (Individual(s) Involved: Self): Research Grant or Support; Scynexis (Individual(s) Involved: Self): Research Grant or Support; SeLux Diagnostics (Individual(s) Involved: Self): Research Grant or Support; Shionogi (Individual(s) Involved: Self): Research Grant or Support; Specific Diagnostics (Individual(s) Involved: Self): Research Grant or Support; Spero (Individual(s) Involved: Self): Research Grant or Support; SuperTrans Medical LT (Individual(s) Involved: Self): Research Grant or Support; T2 Biosystems (Individual(s) Involved: Self): Research Grant or Support; The University of Queensland (Individual(s) Involved: Self): Research Grant or Support; Thermo Fisher Scientific (Individual(s) Involved: Self): Research Grant or Support; Tufts Medical Center (Individual(s) Involved: Self): Research Grant or Support; Universite de Sherbrooke (Individual(s) Involved: Self): Research Grant or Support; University of Iowa (Individual(s) Involved: Self): Research Grant or Support; University of Iowa Hospitals and Clinics (Individual(s) Involved: Self): Research Grant or Support; University of Wisconsin (Individual(s) Involved: Self): Research Grant or Support; UNT System College of Pharmacy (Individual(s) Involved: Self): Research Grant or Support; URMC (Individual(s) Involved: Self): Research Grant or Support; UT Southwestern (Individual(s) Involved: Self): Research Grant or Support; VenatoRx (Individual(s) Involved: Self): Research Grant or Support; Viosera Therapeutics (Individual(s) Involved: Self): Research Grant or Support; Wayne State University (Individual(s) Involved: Self): Research Grant or Support **Lalitagauri M. Deshpande, PhD**, **AbbVie (formerly Allergan**) (Research Grant or Support)**Pfizer, Inc.** (Research Grant or Support) **Timothy B. Doyle**, **AbbVie (formerly Allergan**) (Research Grant or Support)**Bravos Biosciences** (Research Grant or Support)**GlaxoSmithKline** (Research Grant or Support)**Melinta Therapeutics, Inc.** (Research Grant or Support)**Pfizer, Inc.** (Research Grant or Support)**Shionogi** (Research Grant or Support)**Spero Therapeutics** (Research Grant or Support) **Rodrigo E. Mendes, PhD**, **AbbVie** (Research Grant or Support)**AbbVie (formerly Allergan**) (Research Grant or Support)**Cipla Therapeutics** (Research Grant or Support)**Cipla USA Inc.** (Research Grant or Support)**ContraFect Corporation** (Research Grant or Support)**GlaxoSmithKline, LLC** (Research Grant or Support)**Melinta Therapeutics, Inc.** (Research Grant or Support)**Melinta Therapeutics, LLC** (Research Grant or Support)**Nabriva Therapeutics** (Research Grant or Support)**Pfizer, Inc.** (Research Grant or Support)**Shionogi** (Research Grant or Support)**Spero Therapeutics** (Research Grant or Support) **Helio S. Sader, MD, PhD, FIDSA**, **AbbVie (formerly Allergan**) (Research Grant or Support)**Basilea Pharmaceutica International, Ltd.** (Research Grant or Support)**Cipla Therapeutics** (Research Grant or Support)**Cipla USA Inc.** (Research Grant or Support)**Department of Health and Human Services** (Research Grant or Support, Contract no. HHSO100201600002C)**Melinta Therapeutics, LLC** (Research Grant or Support)**Nabriva Therapeutics** (Research Grant or Support)**Pfizer, Inc.** (Research Grant or Support)**Shionogi** (Research Grant or Support)**Spero Therapeutics** (Research Grant or Support)

